# p21^Waf1 ^expression is regulated by nuclear intermediate filament vimentin in neuroblastoma

**DOI:** 10.1186/1471-2407-10-473

**Published:** 2010-09-02

**Authors:** Xénia Mergui, Marie-Line Puiffe, Dominique Valteau-Couanet, Marc Lipinski, Jean Bénard, Mounira Amor-Guéret

**Affiliations:** 1 Université Paris Sud-11, CNRS, UMR 8126, Institut de Cancérologie Gustave Roussy, 114 rue Edouard Vaillant, Villejuif, F-94805, France; 2 Oncopediatric Department, Institut de Cancérologie Gustave Roussy, 114 rue Edouard Vaillant, Villejuif, F-94805, France; 3 Institut Curie, Centre de Recherche, Bâtiment 110, Orsay, F-91405, France; 4 CNRS, UMR 3348, Orsay, F-91405, France

## Abstract

**Background:**

Human neuroblastoma (NB) cell lines may present with either one of the so-called S-and N-subtypes. We have previously reported a strong correlation between protein expression levels of vimentin, an S-subtype marker, and the p21^Waf1 ^cyclin-dependent kinase inhibitor. We here investigated whether this correlation extend to the mRNA level in NB cell lines as well as in patients' tumors. We also further explored the relationship between expression of vimentin and p21, by asking whether vimentin could regulate p21 expression.

**Methods:**

Vimentin and p21 mRNA levels in NB cell lines as well as in patients' tumors (*n *= 77) were quantified using Q-PCR. Q-PCR data obtained from tumors of high risk NB patients (*n *= 40) were analyzed in relation with the overall survival using the Log-rank Kaplan-Meier estimation. siRNA-mediated depletion or overexpression of vimentin in highly or low expressing vimentin cell lines, respectively, followed by protein expression and promoter activation assays were used to assess the role of vimentin in modulating p21 expression.

**Results:**

We extend the significant correlation between vimentin and p21 expression to the mRNA level in NB cell lines as well as in patients' tumors. Overall survival analysis from Q-PCR data obtained from tumors of high risk patients suggests that lower levels of p21 expression could be associated with a poorer outcome. Our data additionally indicate that the correlation observed between p21 and vimentin expression levels results from p21 transcriptional activity being regulated by vimentin. Indeed, downregulating vimentin resulted in a significant decrease in p21 mRNA and protein expression as well as in p21 promoter activity. Conversely, overexpressing vimentin triggered an increase in p21 promoter activity in cells with a nuclear expression of vimentin.

**Conclusion:**

Our results suggest that p21 mRNA tumor expression level could represent a refined prognostic factor for high risk NB patients. Our data also show that vimentin regulates p21 transcription; this is the first demonstration of a gene regulating function for this type III-intermediate filament.

## Background

Neuroblastoma (NB), the most common extracranial solid tumor in children, derives from the sympathetic nervous system. It may follow various courses, from spontaneous regression (stage 4S) to aggressive treatment-resistant diseases, which is often metastatic (stage 4) when diagnosed in children aged 12 months or older. This refractory form predominates over all other forms of NB and has a poor prognosis, with an event-free survival at five years of only 30% [1,2].

NB tumors display various morphologies [1] and NB-derived cell lines are classified into two subtypes on the basis of their appearance and expression profiles, the "N" (Neuronal) and "S" (Substrate-adherent) types [3]. N-type NB cells produce neurofilament proteins and neuron-specific enolase (NSE) but little or no vimentin. Conversely, S-type NB cells produce larger amounts of vimentin than N-type cells [3]. We have previously reported the existence of distinctly altered cellular responses to DNA double-strand breaks (DSB) in "N-type" *versus *"S-type" NB cell lines [4]. In the course of this work, we also noticed that the expression of the type-III intermediate filament vimentin, a marker of S-type cells, correlated with that of the p21^Waf1 ^cyclin-dependent kinase (CDK) inhibitor [4]. Several lines of data support the involvement of vimentin in the regulation of gene expression and in the maintenance of genome stability [5]. Indeed, vimentin, which is known to interact with DNA directly via its N-terminal non-helical domain [6], binds mobile and repetitive sequence elements as well as nuclear matrix attachment regions (MARs) and also displays a strong affinity for four-way junctions [7,8].

We have now verified that the previously reported correlation between expression of vimentin and p21 also existed at the mRNA level in NB cell lines. This allowed us to extend our analysis to tumor samples in which p21 mRNA levels were found associated with clinical evolution. We also report that vimentin partly localizes to the nucleus of NB cells and regulates p21 transcription.

## Methods

### Cell culture, transfections and chemical treatments

Ten NB cell lines derived from patients with advanced stages of disease were used: SK-N-SH, CHP-212, GI-M-EN, CLB-Pe, LAN-5, SJN-B1, CLB-Ga, IGR-N-91, Kelly and IMR32. They were cultured as described [4].

For siRNA transient transfections, cells were either left untreated (Unt.) or submitted to 2 successive transfections at 4 days interval with either siRNA (siCTRL or siVIM, see below) at a 100 nM concentration with Dharmafect 1 (Dharmacon) according to manufacturer's instructions. Four days after the second transfection, cells were treated for 30 min with 200 ng/ml of neocarzinostatin (NCS, Sigma) to induce an ATM-dependent response to DNA double-strand breaks or left untreated. In luciferase assays, cells were transfected with 800 ng of pGL3-luc negative control or pWaf1-luc using the Lipofectamine LTX reagent (Invitrogen), according to manufacturer's instructions.

For overexpression assays, cells were transfected with 1.5 μg of pcDNA3.1 (CTRL) or pcDNA3.1-VIM using Lipofectamine LTX (Invitrogen). For luciferase assays, they were concomitantly transfected with either 500 ng of pGL3-luc or pWaf1-luc.

For the establishment of stable clones, SK-N-SK cells seeded in 10 cm dishes were transfected with 24 μg of the shRNA-VIM containing vector with Lipofectamine 2000 (Invitrogen). Clones were selected under 1 μg/ml puromycin for 14 days.

### Tumor samples

Ethics approval of the study was obtained from the *French Neuroblastome Review Board *(chairman Dr Herbé Rubie, Toulouse, France "rubie.h@chu-toulouse.fr"). According to the form of the neuroblastoma disease and patient age at diagnosis (localized or metastatic), as well as MYCN content, specific National protocols are systematically applied in each referenced french center.

Tumor material from NB patients (*n *= 77) (Additional file 1, Table S1) was obtained at diagnosis at the Institut Gustave Roussy (french center that is referenced for neuroblastoma therapeutic management) with written parents' informed consent. Indeed, in each french center that is referenced for neuroblastoma therapeutic management, before diagnosis statement, parents are informed (written information of pediatric oncologist at the end of 1st consultation), and they are asked for a signed consent of authorizations regarding computerization of data (parents authorization with regards to computerization of data) as well as use of remainders of tumor tissues (parents' authorization with regards to use of tumor tissues that remains from the systematic *MYCN *genome content test for improvement of diagnosis and therapy).

According to the International Neuroblastoma Staging System (INSS), patients were staged as follows: Stage 4 S (*n *= 11), Stage 1 (*n *= 6), Stage 2 (*n *= 10), Stage 3 (*n *= 4) and Stage 4 (*n *= 47, with 6 < 12 months). Total RNAs were extracted and corresponding cDNA obtained as described [9].

### Real-time quantitative PCR analysis

Total mRNA from exponential growing culture of high risk NB cell lines or NB tumor samples were extracted with the RNeasy Mini Kit and the RNeasy Micro Kit (Qiagen). After quantification with a NanoDrop 1000 (Labtech), 1 μg of cell lines mRNA or 200 ng of tumor samples mRNA were converted in cDNA as described previously [4]. Vimentin and p21 mRNA quantification was assessed by Q-PCR with an AbiPrism 7900HT apparatus (Applied Biosystems), in a 25 μl reaction volume containing 12.5 ng of cDNA template, 10 pmol of each primers, and 12.5 μl of Sybr Green Master Mix (Applied Biosystems), with the following oligonucleotides. For vimentin: Vim_F: 5'- AAGAGAACTTTGCCGTTGAA-3' and Vim_R: 5'-GTGATGCTGAGAAGTTTCGT-3'. For p21: p21_F: 5'-GGATGTCCGTCAGAACCCAT-3' and p21_R: 5'- CCCTCCAGTGGTGTCTCGGTG-3'. Normalization was assessed after quantification of GAPDH mRNA or 18 S rRNA levels as indicated with the following oligonucleotides: GAPDH_F: 5'-CTGCACCACCAACTGCTTAG-3' and GAPDH_R: 5'-AGGTCCACCACTGACACGTT-3'; 18S_F: 5'- cggctaccacatccaaggaa-3' and 18S_R: 5'- GCTGGAATTACCCCGGCT-3'.

### siRNAs and constructs

RNA interference experiments were performed using "siVIM" (siGENOME SMART pool for human vimentin, Dharmacon), "siCTRL" (ON-TARGETplus siCONTROL Non-targeting pool, Dharmacon), or the pLKO.1 vector containing the vimentin-targeting shRNA (MISSION shRNA TRCN0000029123, Sigma). The plasmid construct pcDNA3.1-VIM was obtained by cloning the full-length vimentin cDNA (KpnI-Not1) from the pCMV-SPORT6-VIM vector (clone 2985712, Accession Number: BC000163, OpenBiosystems) in the pcDNA3.1 vector (Invitrogen). The pWaf1-luc reported plasmid was kindly provided by Dr May [10]. The pcDNA3.1 (Invitrogen) and pGL3-luc vectors (Promega) were used as negative controls.

### Western Blot analysis

Total cellular extracts or differential fractionation into cytoplasmic and nuclear extracts were performed and submitted to SDS-PAGE as described previously [4]. The primary antibodies used were all monoclonal antibodies and were: anti-vimentin RV202 (1:1000 dilution; Abcam), anti-p21 (F-5; 1:200 dilution; Santa Cruz Biotechnology), anti-α-Tubulin (1:1000 dilution, Sigma), anti-β-actin (1:5000 dilution; Chemicon International Inc.), anti-Topoisomerase IIα (ab45175, 1:20000 dilution; Abcam).

### Immunofluorescence analysis

Cells were seeded on coverslips placed in a 6-wells plate and incubated overnight. Cells were then washed once with PBS, fixed with 4% paraformaldehyde in PBS for 30 min, and rinsed 3 times with PBS. Incubation with cold methanol for 5 min at -20°C was used to permeabilize the cells. Cells were then washed 3 times with PBS and non-specific binding sites were blocked by incubation with PBS-FCS 10% for 30 min. Cells were subsequently incubated with either PBS-BSA 0.1% without any primary antibody, with the mouse anti-Vim antibody RV202 (Figure S2) or with a mix of the goat anti-Vim antibody (1:20 dilution; Sigma) and the mouse anti-Lamin A/C antibody (clone 636; 1:50 dilution; Santa Cruz Biotechnology) (Figure S3). Cells were rinsed 3 times with PBS, and incubated with PBS-BSA 0.1% containing the secondary antibodies at a 1:400 dilution (Alexa 488-conjugated donkey anti-goat, Alexa 647-conjugated chicken anti-mouse, Invitrogen) and cells were then washed 3 times in PBS. Images were taken from a 63X immersion objective of a LSM-510 microscope (Zeiss). z-stacked focal planes presented in the figures are those with the denser/darker DAPI staining, thus corresponding to the inside of nuclei.

### Luciferase reporter assays

For luciferase reporter assays, cells were lysed with Passive Lysis Buffer (50 μl/well) provided with the Luciferase Reporter Assay System (Promega) 24 h after reporter plasmids transfection. Firefly luciferase activity was measured in duplicate with the Luciferase Reporter Assay System (Promega), and luminosity was measured with the Microlumat LB96P apparatus (Berthold EG & G Instrument). The luciferase activity measured after transfection of the pGL3-luc negative control vector was equal to the background in each cell line (not reported).

### Statistical analysis

The Mann-Whitney (luciferase assays) and log-rank statistical tests (overall survival analysis) were performed with the Graph Pad Prism Software version 4.0. The p value cut-off for significance is 0.05.

## Results and Discussion

### Expression levels of vimentin and p21 correlate in NB cell lines and tumor samples with low p21 expression being associated with worse prognosis in high risk patients

**Figure 1:**
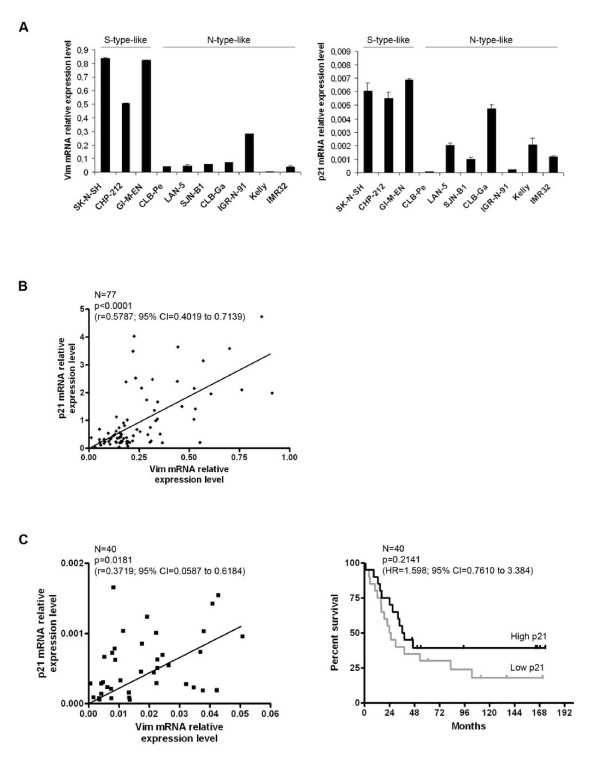
**Vimentin-p21 mRNA correlation in NB cell lines and tumors - Association between p21 expression and clinical evolution**. A. mRNA quantification of vimentin (*left panel*) and p21 (*right panel*) relative expression level after GAPDH normalization, in 10 high risk NB cell lines, using Sybr Green Q-PCR. The S- or N-subtype of the NB cell lines is indicated. This result was confirmed after EF1α normalization and by TaqMan Q-PCR quantification, using two different genes for normalization (18 S and TFRC) (data not shown). Bars and error bars represent the means and standard deviations, respectively. B. Representation of the p21 mRNA relative expression level according to that of vimentin, quantified by Sybr Green Q-PCR after 18 S normalization, in 77 NB tumor samples collected at the time of diagnosis (primary tumor biopsies or metastatic bone marrows, all containing more than 70% of tumor cells) (Clinical data are presented in Additional file 1, Table S1). A cDNA sample from the SK-N-SH cell line was used as a reference to integrate all the data together in a unique analysis. The Spearman statistical test was performed with the Graph Pad Prism Software version 4.0. CI: Confidence Interval. C. *Left panel: *Representation of the p21 mRNA relative expression level according to that of vimentin, quantified by TaqMan Q-PCR after 18 S normalization, in the 40 high risk NB tumor samples of the cohort. The Spearman statistical test was performed as in 1B. This result was obtained using either Sybr-Green or TaqMan technology with two normalization genes, 18 S and GAPDH, or 18 S and TFRC, respectively (data not shown). *Right panel: *The survival analysis of patients older than one year of age with a stage 4 NB (N = 40) was performed using the Log-rank Kaplan-Meier estimation with the Graph Pad Prism Software version 4.0. The overall survival was defined as the time in months between diagnosis and death or last follow-up (excluding patients who died from toxicity). Patients were separated into two distinct groups according to their p21 mRNA tumor relative expression level quantified by Q-PCR as in the left panel. The median of p21 tumor expression was chosen as a cut-off between high (> median, black line) and low (< median, grey line) expression. HR: Hazard Ratio, CI: Confidence Interval.

We initially observed that levels of vimentin and p21 were correlated at the protein level in S-type-like NB cell lines [4]. To test whether this was also true at the mRNA level, ten NB cell lines were tested by real-time quantitative PCR (Q-PCR). As seen in Figure 1A, the three S-type-like cell lines expressed high levels of vimentin and p21 mRNAs. Although more heterogeneous, expression levels were consistently lower in the six N-type-like cell lines tested.

We then measured mRNA levels for both vimentin and p21 in NB tumor samples obtained at diagnosis from 77 children (all stages; see Additional file 1: Table S1). We found a highly significant correlation between the mRNA levels of the two genes (Spearman *r = *0.5787, 95% confidence interval (CI) = 0.4019 to 0.7139, p < 0.0001) (Figure 1B). Although weaker, this correlation held true when the 40 stage 4 NB tumor samples were considered separately (Spearman *r *= 0.3719, 95% CI = 0.0587 to 0.6184, p = 0.0181) (Figure 1C, *left panel*). We thus conclude that vimentin and p21 mRNA levels are correlated in both NB cell lines and tumor samples.

Loss of p21 expression has been reported to be a poor prognostic factor in colorectal carcinomas and small-cell lung cancers [11]. In the presently used NB cohort, no statistically significant association was identified between p21 mRNA levels in tumor cells and any available clinical data (age at diagnosis, INSS stage, *MYCN *amplification status) (data not shown). Many reports have indicated that clinical and genomic tumor criteria allow a prognostic classification of NB [12,13]. However, no stratifying prognostic factor has been proposed so far for high risk NB (MYCN-amplified and stage 4 NB of patients > 12 months). We here analyzed p21 tumor expression levels in relation with the overall survival (OS) of the 40 high risk NB patients. Despite the limited size of the cohort, two groups of patients were identified using the Log-rank Kaplan-Meier estimation (Figure 1C, *right panel*) that appeared to exhibit different survival curves according to the "high" or "low" p21 mRNA expression levels in the corresponding tumors (p = 0.2141, Hazard Ratio (HR) = 1.598, 95% CI = 0.7610 to 3.384), the median tumor p21 mRNA level of the cohort being chosen as the cut-off between high (> median) and low (< median) levels of expression. The patients with low p21 tumor expression at diagnosis had a 24.6 months median survival compared with 37.2 in high p21 tumor expressors.

We analyzed the relationship between p21 mRNA expression in stage 4 NB tumors and overall survival using microarray gene expression data from the R2 repository http://hgserver1.amc.nl/cgi-bin/r2/main.cgi. While the p21 probe set #202284 did not give significant results, the p21 probe set #1555186 revealed that low levels of p21 mRNA are highly significantly associated with a reduced OS (data not shown), consistent with the tendency we observed.

Altogether, these results suggest that our finding would be of great interest to orientate therapeutic decisions, if confirmed in larger groups of high risk NB patients.

### Vimentin participates in p21 transcriptional regulation

**Figure 2:**
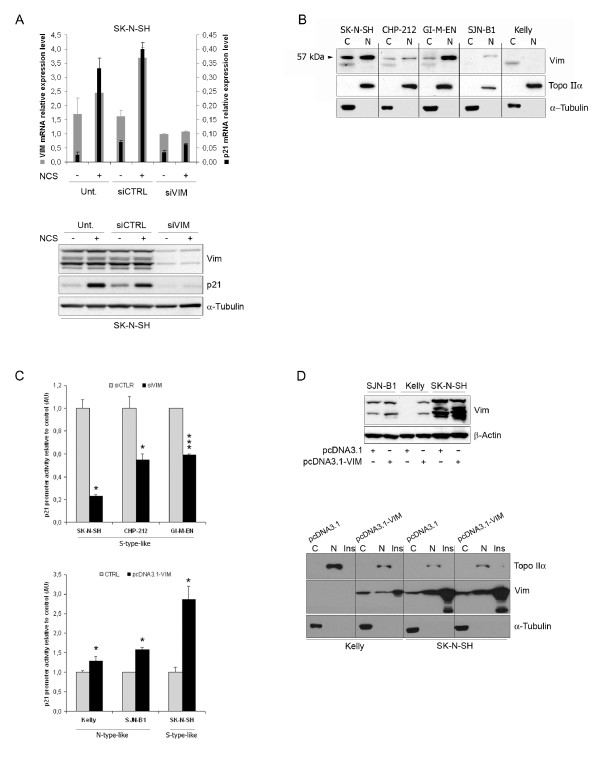
**Vimentin participates in p21 transcriptional regulation**. A. Vimentin or p21 gene expression was monitored by Q-PCR analysis (*upper panel*), as described in Figure 1A. Vimentin or p21 protein levels in total cellular extracts were assessed by immunoblotting, α-Tubulin being used as loading control (*lower panel*). Cells were left untransfected (Unt.) or were transfected with the siRNAs targeting vimentin (siVIM) or control siRNA (siCTRL). Cells were either untreated (-) or NCS-treated (+). This figure is representative of 3 independent experiments. NCS: neocarzinostatin. B. Subcellular localization of soluble vimentin assessed by differential fractionation (C, Cytoplasmic; N, nuclear) followed by Western blot analysis in SK-N-SH, CHP-212, GI-MEN, SJN-B1 and Kelly cells lines. Topoisomerase IIα and α-Tubulin are used as controls for the nuclear and cytoplasmic soluble fractions, respectively. C. Analysis of p21 promoter activity using luciferase assay in indicated S- or N-type-like NB cell lines, either downregulated (siVIM) or not (siCTRL) for vimentin expression *(upper panel)*, or overexpressing (pcDNA3.1-VIM) or not (CTRL: pcDNA3.1) vimentin *(lower panel)*. Comparisons were assessed with the Mann-Whitney statistical test (* and *** stand for p < 0.05 and p < 0.005 respectively). The graph is representative of 3 independent experiments. D. *Upper panel: *Western Blot analysis of vimentin expression in indicated cells transiently transfected with pcDNA3.1-VIM or the empty vector (pcDNA3.1). β-actin is used as loading control. *Lower panel: *Vimentin cellular distribution (C, Cytoplasmic soluble; N, nuclear soluble; Ins., Insoluble cytoskeletal/matrix-associated fractions) in vimentin overexpressing cells, assessed by differential cellular fractionation followed by Western Blot analysis. Topoisomerase IIα and α-Tubulin are used as controls for the nuclear and cytoplasmic soluble fractions, respectively.

To further explore the relationship between expression of vimentin and p21, we then asked whether vimentin could regulate p21 expression. A pool of siRNAs was used to inhibit vimentin expression in the SK-N-SH S-type-like NB cell line (Figure 2A). First of all, it is worth noting that vimentin depletion was not accompanied by any cellular morphological changes (data not shown). Q-PCR analysis (*upper panel*) revealed that p21 and vimentin mRNA levels decreased concomitantly. Whereas p21 was strongly induced in vimentin expressing control cells whose DNA was damaged following NCS treatment, cells inhibited for vimentin expression using siRNAs proved unresponsive to NCS with hardly any increase in p21 expression (*lower panel*). We also generated SK-N-SH clones stably expressing a shRNA directed against a vimentin mRNA sequence different from that targeted by the siRNAs. Four SK-N-SH shRNA-Vim clones were selected with various protein expression levels of vimentin. Strikingly, p21 protein levels were found to strictly correlate with those of vimentin (*r *= 0.97, see Additional file 2, Figure S1). Altogether, these results suggest that vimentin may directly or indirectly regulate p21 expression.

As vimentin has been reported to bind nucleic acids [7,14], we then used confocal immunofluorescence microscopy to determine the cellular distribution of vimentin in NB cell lines. The two cell lines strongly expressing vimentin, SK-N-SH and GI-M-EN, displayed much stronger staining than the SJN-B1 and Kelly cell lines, which produce little or no vimentin, respectively (see Additional file 2, Figure S2). In addition to the classical dense cytoplasmic staining for filamentous vimentin, we observed weak but nonetheless evident staining for vimentin in the nucleus of SK-N-SH and GI-M-EN cells (see Additional file 2, Figure S2). We also observed some interesting colocalization of vimentin with the nuclear matrix lamin A/C protein on the inner side of the nuclear membrane (see Additional file 3, Figure S3). We then used a biochemical approach for the detection of nuclear and cytoplasmic soluble proteins by immunoblotting. Soluble vimentin was detected in nuclear extracts in all three cell lines strongly expressing vimentin (SK-N-SH, CHP-212, GI-M-EN), and, to a lower extent, in the SJN-B1 cell line, which contained smaller amounts of vimentin, but no soluble vimentin was detected in the nucleus of the vimentin-negative Kelly cell line (Figure 2B). However, we cannot formally exclude the potential binding of vimentin to the outside of the nucleus that could produce its inclusion in the nuclear fraction.

Being potentially partly localized in the nucleus, vimentin may well behave like a bona fide transcription factor in these NB cells. To test this hypothesis, the SK-N-SH, CHP-212 or GI-M-EN cell lines which express high levels of p21 were transfected with plasmid vectors containing the luciferase reporter gene downstream from either 2400 bp of the p21 gene promoter (pWaf1-luc; [10]) or a minimal gene promoter used as a control (pGL3-luc). In all three cell lines, p21 promoter activity depended upon the expression of vimentin as demonstrated using a vimentin siRNAs pool in comparison with control siRNAs (Figure 2C, *upper panel*). Conversely, co-transfecting the luciferase reporter vectors along with a plasmid directing vimentin expression demonstrated that the transient overexpression of vimentin induced a significant, but moderate, increase in p21 promoter activity in vimentin-negative or low-expressing cell lines Kelly and SJN-B1, respectively. The inducing effect was even stronger in the already high vimentin-expressing cell line SK-N-SH (Figure 2C, *lower panel*). Transfection efficiency was similar (approximately 35% of cells transfected, data not shown) in all three cell lines as revealed by flow cytometry analysis of cells co-transfected with a GFP expression vector and the pcDNA3.1-VIM plasmid. That vimentin was indeed overexpressed in all three cell lines, albeit to different extents, was demonstrated by immunoblotting analysis shown in Figure 2D (*upper panel*). Finally, the vimentin negative Kelly cell line and the SK-N-SH cell line which displayed high levels of vimentin were transiently transfected with an empty vector or with a construct encoding vimentin. We then fractionated the cells into cytoplasmic soluble, nuclear soluble and cytoskeletal/matrix-associated insoluble fractions. In all cases where vimentin was expressed (at the basal level or after transfection), most of it was detected in the insoluble fraction, as expected for the cytoplasmic intermediate filament form of the protein (Figure 2D, *lower panel*), but some vimentin was also detected in the nuclear soluble fraction.

From our results, we conclude that vimentin regulates p21 gene transcription, directly or indirectly, through promoter activation. Indeed, it remains unclear whether vimentin regulates p21 gene transcription through direct or indirect effects on its promoter, and further experiments are required to address this issue. Vimentin could have an indirect effect by modifying the chromatin organization of the promoter, as suggested by its specific binding to chromatin-modifying proteins, such as the HBO1 histone acetyltransferase or the SETDB1 histone methyltransferase, by acting as transcription cofactor, as suggested by its binding to the PIASγ transcription regulator [15], or by modifying the global organization of the nucleus, as we observed colocalization with the lamin A/C proteins known to regulate nuclear organization [16].

Cytoskeletal proteins thus appear to be endowed with dual functions, both in maintaining cell architecture and in regulating gene expression. Actin has been known for some years to regulate transcription [17]. More recently, some lamins have been reported to perform similar function [18] but to our knowledge, there has been no previous demonstration of a gene regulating function for vimentin.

In the context of neuroblastoma oncogenesis, the expression of the vimentin and p21 genes is regulated during neuronal differentiation. The vimentin gene is expressed early in neural crest cells development [19] and is involved in initiating neurite outgrowth [20,21], and p21 is also known to be induced during neural development [22]. Strong vimentin and p21 expression in some tumors may indicate that oncogenesis occurred at a specific time point in neural crest cell development. Low or no vimentin and p21 in other tumors may indicate that oncogenesis took place at a different stage of differentiation of the tumor progenitors.

## Conclusion

Here, we show a significant correlation between expression of vimentin and p21 at the mRNA level in NB cell lines and patients' tumors. Overall survival analysis based on tumors of high risk patients suggest that lower levels of p21 expression could be associated with a poorer outcome. We additionally report that vimentin partly localizes to the nucleus of NB cells and regulates p21 transcription.

## List of abbreviations

NB: neuroblastoma; DSB: double-strand break; CDK: cyclin-dependent kinase; NCS: neocarzinostatin.

## Competing interests

The authors declare that they have no competing interests.

## Authors' contributions

Conceived and designed the experiments: XM, MAG. Performed the experiments: XM, MLP. Analyzed the data: XM, MAG. Contributed reagents/materials/analysis tools: JB, MAG, DVC, ML. Wrote the paper: XM, MAG, ML. All authors read and approved the final manuscript.

## Pre-publication history

The pre-publication history for this paper can be accessed here:

http://www.biomedcentral.com/1471-2407/10/473/prepub

## Supplementary Material

Additional file 1 **Table S1**. Available clinical data for the *n *= 77 NB patients of the studied cohort (INSS Stage, sex and age of the patient, MYCN amplification status, patient status and follow-up). M: male, F: female, NA: MYCN non-amplified, A: MYCN amplified, A: alive, D: deceased. Click here for file

Additional file 2 **Figure S1 and Figure S2**. Figure S1: Vimentin and p21 protein expression levels assessed by immunoblotting in four SK-N-SH clones selected for the stable expression of a construct expressing a shRNA specific for vimentin. Figure S2: Immunofluorescence staining of vimentin in the SK-N-SH, CHP-212, GI-M-EN, SJN-B1 and Kelly cell lines, analyzed by confocal microscopy. Red: Vimentin; Blue: DAPI. Click here for file

Additional file 3 **Figure S3**. Immunofluorescence staining of vimentin and lamin A/C proteins, in the GI-M-EN, SK-N-SH and CHP-212 cell lines, analyzed by confocal microscopy. Red: Vimentin; Green: Lamin A/C. Click here for file
