# Supplementary figures and images for: p21Waf1 expression is regulated by nuclear intermediate filament vimentin in neuroblastoma
Source: BMC Cancer. 2010 Sep 2;10:473. doi: 10.1186/1471-2407-10-473 (PMC2939553; doi:10.1186/1471-2407-10-473)

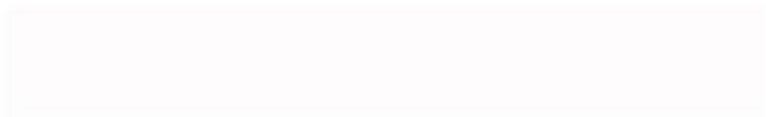

Figure S1

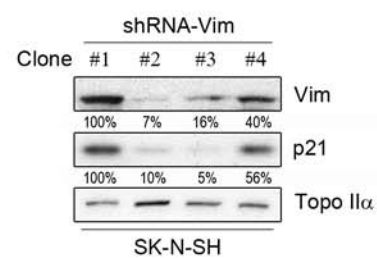

Figure S2

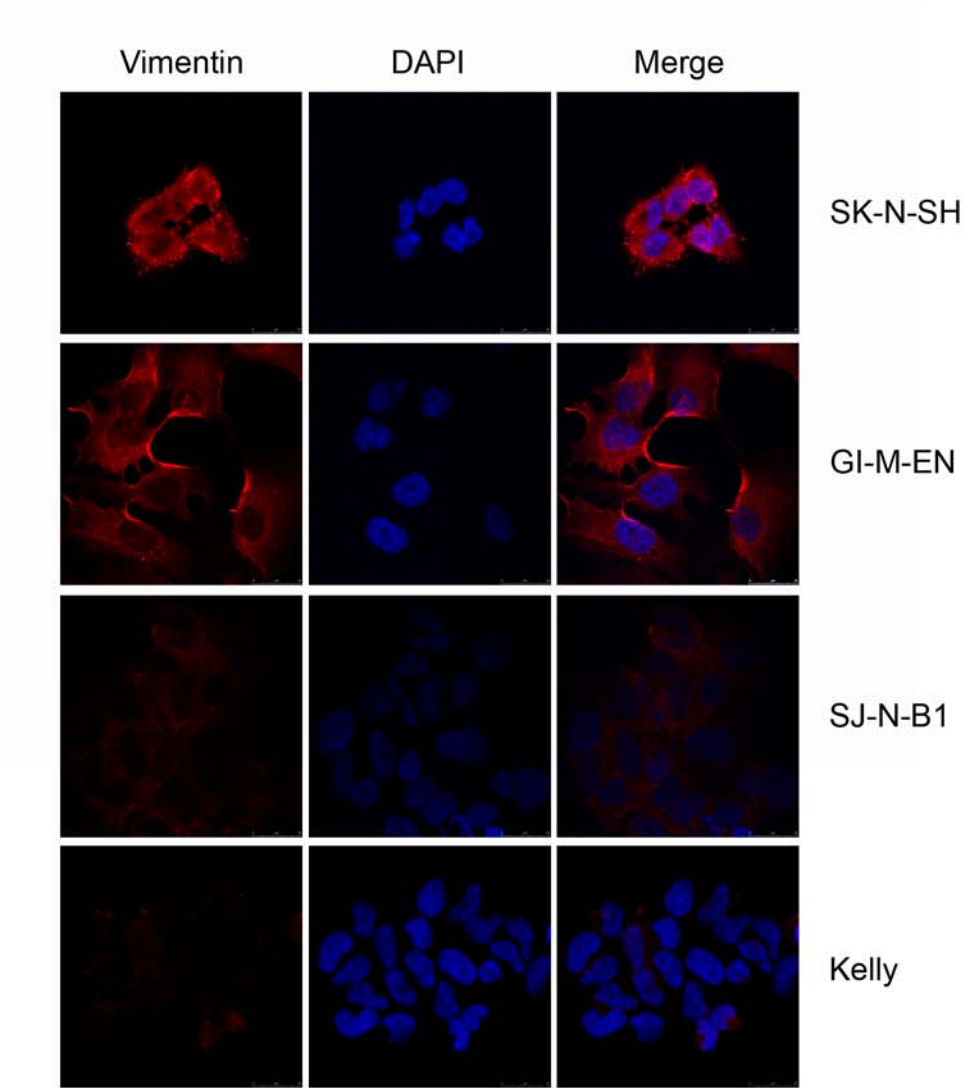

Supplement: Additional file 2 — Figure S1 and Figure S2. Figure S1: Vimentin and p21 protein expression levels assessed by immunoblotting in four SK-N-SH clones selected for the stable expression of a construct expressing a shRNA specific for vimentin. Figure S2: Immunofluorescence staining of vimentin in the SK-N-SH, CHP-212, GI-M-EN, SJN-B1 and Kelly cell lines, analyzed by confocal microscopy. Red: Vimentin; Blue: DAPI. [file 1471-2407-10-473-S2.PDF]

Figure S3

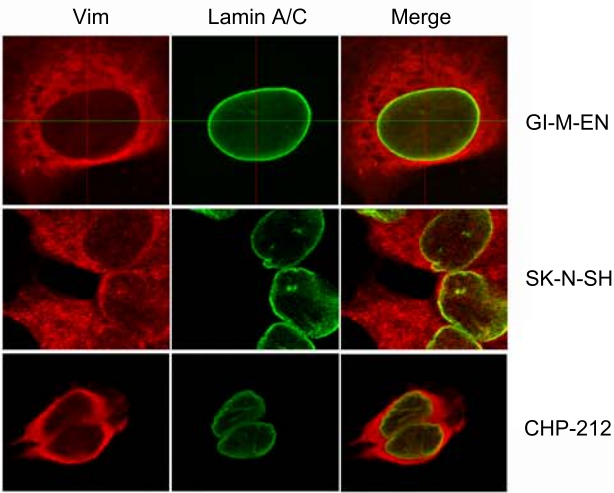

Supplement: Additional file 3 — Figure S3. Immunofluorescence staining of vimentin and lamin A/C proteins, in the GI-M-EN, SK-N-SH and CHP-212 cell lines, analyzed by confocal microscopy. Red: Vimentin; Green: Lamin A/C. [file 1471-2407-10-473-S3.PDF]
